# Moderate-intensity exercise with blood flow restriction on cardiopulmonary kinetics and efficiency during a subsequent high-intensity exercise in young women: A cross-sectional study

**DOI:** 10.1097/MD.0000000000025368

**Published:** 2021-08-06

**Authors:** Robson F. Borges, Gaspar R. Chiappa, Paulo T. Muller, Alexandra Correa Gervazoni Balbuena de Lima, Lawrence Patrick Cahalin, Graziella França Bernardelli Cipriano, Gerson Cipriano

**Affiliations:** a Physical Education Graduate Program, University of Brasilia (UnB), Brasilia, DF, Brazil; b Graduate Program in Human Movement and Rehabilitation of Evangelical University of Goiás, Brazil; c Laboratory of Respiratory Pathophysiology (LAFIR), Federal University of Mato Grosso do Sul, Campo Grande, MS, Brazil; d Health Sciences and Technologies Graduate Program, University of Brasilia (UnB), Brasilia, DF, Brazil; e Department of Physical Therapy, University of Miami Miller School of Medicine, Coral Gables, FL.

**Keywords:** exercise, exercise testing, mathematical modeling, oxygen cost, oxygen kinetics, performance

## Abstract

Blood flow restriction (BFR) training applied prior to a subsequent exercise has been used as a method to induce changes in oxygen uptake pulmonary kinetics ($\dot{\text{V}}$O_2P_) and exercise performance. However, the effects of a moderate-intensity training associated with BFR on a subsequent high-intensity exercise on $\dot{\text{V}}$O_2P_ and cardiac output (Q_T_) kinetics, exercise tolerance, and efficiency remain unknown.

This prospective physiologic study was performed at the Exercise Physiology Lab, University of Brasilia. Ten healthy females (mean ± SD values: age = 21.3 ± 2.2 years; height = 1.6 ± 0.07 m, and weight = 55.6 ± 8.8 kg) underwent moderate-intensity training associated with or without BFR for 6 minutes prior to a maximal high-intensity exercise bout. $\dot{\text{V}}$O_2P_, heart rate, and Q_T_ kinetics and gross efficiency were obtained during the high-intensity constant workload exercise test.

No differences were observed in $\dot{\text{V}}$O_2P_, heart rate, and Q_T_ kinetics in the subsequent high-intensity exercise following BFR training. However, exercise tolerance and gross efficiency were significantly greater after BFR (220 ± 45 vs 136 ± 30 seconds; *P* < .05, and 32.8 ± 6.3 vs 27.1 ± 5.4%; *P* < .05, respectively), which also resulted in lower oxygen cost (1382 ± 227 vs 1695 ± 305 mL min^–1^).

We concluded that moderate-intensity BFR training implemented prior to a high-intensity protocol did not accelerate subsequent $\dot{\text{V}}$O_2P_ and Q_T_ kinetics, but it has the potential to improve both exercise tolerance and work efficiency at high workloads.

## 1 Introduction

Studies have demonstrated that the magnitude of oxygen uptake pulmonary kinetics ($\dot{\text{V}}$O_2P_) adjustments toward a steady-state may be altered by a prior “priming exercise (PE)”.^[1,2]^ The increases in bulk O_2_ delivery to the exercising muscles elicited from a PE bout have dramatic effects on the response to subsequent exercises. Current evidence suggests that a bout of moderate-intensity PE acutely accelerates $\dot{\text{V}}$O_2P_ kinetics in the subsequent training,^[3,4]^ while increasing peripheral muscle blood flow,^[5]^ O_2_ extraction,^[6]^ as well as enzymatic oxidative^[7]^ and electromyographic activity.^[6]^ In general, these results have shown an increased amplitude of the O_2_ primary component, and a reduction in the O_2_ slow component, although with a modest benefit to O_2_ economy.^[8]^

It is known that the effectiveness of a preceding exercise on a subsequent bout of exercise may be influenced by several factors, including training intensity, rest interval between each exercise, and the type of exercise performed.^[6]^ Among these factors, high-intensity training implemented prior to a subsequent bout of exercise seems to increase the performance of the latter due to higher O_2_ availability and acceleration.^[8]^

Furthermore, studies have likewise suggested blood flow restriction (BFR) training or remote ischemic preconditioning (RIPC) as PE methods to optimize performance in several sports due to the resulted increase in blood flow to the limbs during a subsequent exercise,^[9]^ with potential to reduce muscle fatigue,^[10]^ through improvements in oxygen uptake ($\dot{\text{V}}$O_2_) and lower blood lactate accumulation.^[11]^ However, reports of the favorable effects of RIPC or BFR on exercise performance^[12]^ or kinetics^[9]^ appear not to be consistent in the scientific literature. The benefits of RIPC on muscle oxygen kinetics are possibly linked to faster muscle deoxygenation kinetics and an increased $\dot{\text{V}}$O_2_ peak, which has been observed during both moderate and maximal exercise.^[13]^

More recently, it has been suggested that tailoring the priming activity is the key to producing beneficial results.^[14]^ A recent study showed that both low-intensity aerobic exercises associated with BFR and high-intensity aerobic training with no vascular occlusion demonstrated similar effects, increasing oxidative muscle capacity and speeding of $\dot{\text{V}}$O_2_ kinetics.^[15]^ Likewise, despite improving both the acute magnitude of muscular activation and fatigue, a resistance PE performed at low-intensity with vascular occlusion yielded a less profound impact on neuromuscular function than high-intensity resistance exercise training without BFR.^[16]^

Accelerated oxygen uptake capability has been related to several clinical outcomes, including higher physical performance^[17]^ and efficiency.^[18]^ However, the effects of a PE protocol associated with BFR on the pulmonary and cardiovascular kinetics components, as well as exercise tolerance and gross efficiency, have not yet been examined. The rationale for applying PE with BFR is based on previous studies that demonstrated benefits from a high-intensity warm-up but not from a moderate-intensity priming activity on oxygen kinetics. Given that BFR training is considered a method to mimic high-intensity exercise using lower intensity protocols, we believe that the addition of BFR to moderate-intensity exercise can produce benefits which would otherwise be achieved only at higher loads.^[9]^

Thus, the purpose of this study was to examine the effects of a prior bout of moderate-intensity exercise with BFR on $\dot{\text{V}}$O_2P_ and cardiac output (Q_T_) kinetics, exercise tolerance, and work efficiency in young female subjects during a subsequent high-intensity exercise. We hypothesized that faster $\dot{\text{V}}$O_2P_ and Q_T_ kinetics, as well as greater exercise tolerance and gross efficiency would be observed during high-intensity exercise following PE associated with BFR when compared to no PE being implemented.

## 2 Materials and methods

### 2.1 Study design and settings

This cross-sectional study was designed following the STROBE statement.^[19]^ We recruited healthy female subjects, aged ≥50 years from cardiology clinics affiliated with University of Brasília using a convenience sample. Subjects were referred to the Laboratory of Physiology (University of Brasilia, Brazil) from May to July 2018.

### 2.2 Participants

Ten healthy females (mean ± SD values: age = 21.3 ± 2.2 years; height = 1.6 ± 0.07 m, and weight = 55.6 ± 8.8 kg) volunteered to participate in the study. Subjects presented a peak $\dot{\text{V}}$O_2_ (oxygen uptake) of 1569.1 ± 240.0 magnitude mL min^–1^ assessed in a previous incremental ramp exercise test on a cycle ergometer.

Subjects were free from overt cardiovascular, respiratory, musculoskeletal, or metabolic diseases (i.e., no heart or breathing pathology, no diabetes, no chronic medical condition) and were not receiving any prescribed medications prior to study entry. They were non-smokers and presented normal weight (body mass index < 30 kg . m^–2^). All participants underwent a comprehensive pre-test clinical evaluation, including spirometry, as well as resting and exercise electrocardiograms. All participants were instructed not to perform heavy exercise or consume any food or drink that contained caffeine for 72 hours prior to testing. A daily questionnaire was applied to assure compliance with instructions. Subjects were physically active but not engaged in any specific high-intensity exercise training regimens.

### 2.3 Ethical review

Subjects were informed of all discomforts and risks involved with study participation, and their written consent was obtained prior to the study. The experimental protocol was approved by the Medical Ethics Committee of the University of Brasilia (CAAE 50414115.4.0000.0030). All experiments were performed in accordance with relevant guidelines and regulations. The informed consent was obtained from all participants.

### 2.4 Experimental approach

Participants performed an incremental ramp exercise test (10–20 W/min) (visit 1) to determine parameters of aerobic function during exercise. The tests were always performed in the morning, at the laboratory with temperature kept at 22 ± 1.2 °C. These tests were performed on an electronically braked cycle ergometer (Corival 400, Lode, Netherlands) at 60 rpm. The difference between $\dot{\text{V}}$O_2P_ at the gas exchange threshold (GET) ($\dot{\text{V}}$O_2PGET_) and $\dot{\text{V}}$O_2P_ at peak exercise ($\dot{\text{V}}$O_2peak_) (Δ$\dot{\text{V}}$O_2P__peak-GET_) was determined from the ramp exercise test and used to determine the bike load in the subsequent constant-load tests.

**Figure 1:**
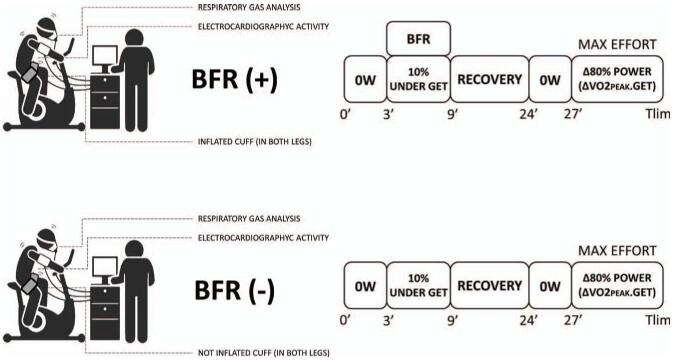
Study design protocol. Demonstration of the data acquired during the tests and the timeline of the constant load tests. BFR = blood flow restriction, 0W = indicate the phase with no load, GET = gas exchange threshold, T*lim* = time limit.

Before starting their training regimen, subjects visited the lab to familiarize themselves with the study protocol (visit 2). On other two separate days (visits 3 and 4; 48-interval between study visits), subjects performed the constant work rate (CWR) exercise tests (Fig. 1). The first test (trial 1) was performed following an initial bout of PE at a moderate-intensity CWR (below the $\dot{\text{V}}$O_2PGET_) randomly associated with (intervention) or without (placebo) BFR for 6 minutes, followed by a maximal high-intensity CWR exercise test (above $\dot{\text{V}}$O_2PGET_), until their limit of tolerance (see Fig. 1). The second test (trial 2) was applied after a 15-minute recovery period and preceded by unloaded baseline pedaling at 0 W for 3 minutes. The examiner who implemented the protocol associated with vascular occlusion was different from the one who applied the other exercises. Studies have used different rest intervals between priming and subsequent training bouts, with time periods ranging from 7 to 15 minutes.^[9]^ We decided to use a 15-minute interval between training bouts as it was considered an adequate period to ensure a proper heart rate return to baseline before starting the high-intensity protocol in order to avoid any interference when it comes to the heart rate (HR) kinetics analysis.

### 2.5 Blood flow restriction

During the intervention phase of the protocol, the vascular occlusion was performed using calibrated blood pressure sphygmomanometers, with large cuffs, for circumferences of 35 to 51 cm (Classic Hand Aneroid with Adult Cuff, Welch Allyn, USA). The cuffs were placed around the most proximal parts of the legs and loosely kept in place with elastic bandages throughout the prior exercise bout. The cuffs were inflated to 130% of the baseline systolic blood pressure (SBP) bilaterally, approximately 10 seconds before the CWR exercise test, and deflated immediately after 6 minutes of exercise to produce faster reperfusion, in accordance with previously described methods.^[20]^ During the placebo phase of the protocol, the cuffs were positioned similarly to the intervention group but remained deflated throughout the exercise.

### 2.6 Outcome measurements

#### 2.6.1 Incremental ramp exercise test

Symptom-limited cycle ergometry exercise tests were performed using a computer-based exercise system (Quark CPET, Cosmed, Roma, Italy) with breath-by-breath analysis of metabolic, ventilatory, and cardiovascular variables. The following data were recorded at mean intervals of 10 seconds: oxygen uptake ($\dot{\text{V}}$O_2_, mL/min), minute ventilation ($\dot{\text{V}}$E, L/min), respiratory rate (rpm), and tidal volume (L).

The $\dot{\text{V}}$O_2P_ GET was estimated through the gas-exchange method, visually inspecting the inflection point of $\dot{\text{V}}$CO_2P_ with regard to $\dot{\text{V}}$O_2P_ (modified V-slope)^[13]^ and through the ventilatory method when the VE-to-$\dot{\text{V}}$O_2P_ ratio and end-tidal partial pressure of O_2_ increased, while the VE-to-$\dot{\text{V}}$CO_2p_ ratio and end-tidal partial pressure of CO_2_ remained stable. The reading was performed independently by two experienced observers who were blinded to what intervention participants received and their identities. Subjects were also asked to rate their “shortness of breath” at exercise cessation using the 0–10 Borg category-ratio scale: symptom scores were expressed in absolute values.

#### 2.6.2 Constant work rate exercise test

As previously described,^[13]^ the CWR tests were preceded by a 2-minute unloaded warm-up at 60 rpm, followed by a stepwise increase in WR to 80% of Δ$\dot{\text{V}}$O_2_peak, above the LT and until limit of tolerance. After the exercise test, exhaled gas data were obtained for 10 minutes. The limit of tolerance was defined as the time point at which the subjects signaled to stop exercising or could not maintain the required pedaling rate for 10 seconds, despite being encouraged by the investigators.^[13]^

#### 2.6.3 Cardiovascular measures

Cardiac output (Q_T_, L/min) and stroke volume (L) were estimated non-invasively, using the indirect Fick method. The cardiac output was calculated using the following equation:

(1) $${\text{Q}}_{\text{T}}=\text{Ejection}\;\text{volume}\;\times \text{heart}\;\text{rate}$$

Fick principle has been applied to estimate cardiac output and involves calculating the oxygen consumption over a given period using measurements of venous and arterial blood. Both heart rate and O_2_ consumption can be easily measured during an incremental cardiopulmonary exercise test, thereby cardiac output and ejection volume can be precisely quantified if the arteriovenous difference is estimated. This arteriovenous difference demonstrates a linear increase as a function of the percentage of $\dot{\text{V}}$O_2_ max in healthy subjects. Assuming this relationship and considering the Fick equation, some references demonstrate that it is possible to estimate the cardiac output non-invasively during exercise using this method.^[21]^

### 2.7 Kinetics analysis

The breath-by-breath $\dot{\text{V}}$O_2P_ and cardiovascular (Q_T_ and HR) measures were initially screened for aberrant outliers owing to sighs, coughs, or unexpected respiratory swings; these were eliminated when larger than 4SD from local media.^[22]^ The remaining data were interpolated second by second before kinetics analysis (SigmaPlot 12.5, Systat Software, San Jose, CA) and re-constructed with 3s-bins. We opted to fit $\dot{\text{V}}$O_2P_, Q_T_, and HR data from 30 seconds of baseline pedaling toward a 6-minute bout or maximal attainable exercise, after the onset of exercise (CWR below-AT and high-AT).

A computational Levenberg-Marquardt regression algorithm was performed, constricted to 400 interactions. Our analysis strategy combined an initial plot of residual distribution, inspecting for visual flatness around “zero” after a monoexponential fitting. In the case of a local deviation from “zero”, denoting a slow component, a bi-exponential term was applied in the corresponding data to determine the limits between phases II and III^[23]^:

(2) $$Y(t)=Yb+Ap*(1-e^{-(t-TDp/\tau p)})+Asc*(1-e^{-(t-TDsc/\tau sc)})$$

Where [Y] is the variable under analysis; the subscripts b, p, and sc are baseline unloaded cycling, the primary component, and slow component, respectively; A is the amplitude, TD is the time delay, and **τ** is the time constant of the exponential response of interest. In the case of flatness of the residual plot and absence of a visible slow component, the second term of Eq. (2) was ignored.^[24]^

In the analysis of Q_T_ and HR, the TD_P_ was dropped from the equation. For $\dot{\text{V}}$O_2P_ analysis, we deleted the data relative to the first 20 seconds after exercise onset, the cardio-dynamic phase.^[25]^ Therefore, **τ**$\dot{\text{V}}$O_2P_ represents the time course of the primary component of the response, an estimate of the muscle $\dot{\text{V}}$O_2P_ kinetics.^[26]^

### 2.8 Gross efficiency

Gross efficiency (GE) was calculated as the ratio of work accomplished min^–1^ (i.e., watts converted to kcal min^–1^) to energy expended min^–1^ (kcal min^–1^). Energy expenditure min^–1^ (i.e., kcal min^–1^) was calculated from VO_2_ and RER.^[27]^ Therefore, GE was calculated as the average GE in the final 60 seconds.^[28]^

### 2.9 Statistical analysis

Data analyses were performed using SigmaPlot (Chicago, IL). The results are presented as mean ± standard deviation or median and changes (minimum and maximum). To compare baseline measurements, the Student *t* test for paired samples was utilized. The one-way ANOVA (repeated measures) was used to evaluate outcomes with Bonferroni post-hoc analysis. Subsequently, a mixed model ANOVA with factors of condition (BFR and without BFR sequences, within-subjects) and test order (Trial 1 and Trial 2, between-subjects) was used for the performance data. The adopted significance level was set at *P* ≤ .05 for all tests. The sample size was calculated for moderate effect size (*ES* = 0.5), with an α level of 0.05 and a statistical power of 80%, resulting in ten subjects. The power was calculated according to the primary outcomes (MRT $\dot{\text{V}}$O_2P_, ET, and GE) using a post-hoc analysis in G∗Power software (version 3.1.3; University of Trier, Trier, Germany), considering α = 0.05 and the calculated effect size (d) for each outcome: $\dot{\text{V}}$O_2P_MRT (d: 0.64, power: 57%), ET (d: 2.19, power: 99%), and GE (d: 0.97, power: 80%).

## 3 Results

### 3.1 Subject characteristics

Baseline characteristics of the subjects at rest are presented in Table 1. During the maximal cardiopulmonary test, the subjects obtained a $\dot{\text{V}}$O_2_ of 1569.1 ± 240.0 mL/min, which is compatible with a sedentary sample.^[29]^

**Table 1:** Baseline characteristics of healthy subjects’ women (n = 10).

Variables	Values
Demographic/Anthropometric	
Age (years)	21.3 ± 2.2
Weight (kg)	55.6 ± 8.8
Height (m)	1.6 ± 0.07
BMI (kg/m^2^)	21.2 ± 2.2
Incremental exercise test	
$\dot{\text{V}}$O_2__peak_ (mL/min)	1569.1 ± 24
$\dot{\text{V}}$O_2__peak_ (mL/kg min^–1^)	28.5 ± 4.2
$\dot{\text{V}}$CO_2__peak_ (mL/min)	2191.2 ± 673.8
RER _peak_	1.4 ± 0.1
$\dot{\text{V}}$E _peak_ (L/min)	69.1 ± 23.5
HR _peak_ (bmp)	198.7 ± 56.9
Heart rate (% pred)	85.8 ± 14.0
Power _peak_ (W)	163 ± 21.4

### 3.2 Cardiovascular and pulmonary adjustments

**Figure 2:**
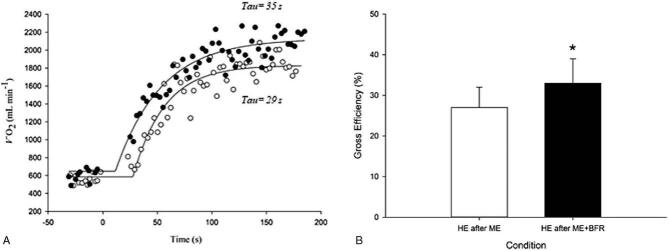
(A) Pulmonary O_2_ uptake ($\dot{\text{V}}$O_2P_) kinetics (phase II) at the onset of heavy-intensity exercise with BFR and without BFR in a representative participant. Closed circle represents high-intensity bout following BFR and open circle high-intensity bout without BFR, *P* > .05. (B) Gross efficiency (GE) during heavy-intensity exercise in a representative participant. Note, the GE was significantly higher after the BFR condition. BFR = blood flow restricted, HE = high-exercise intensity, ME = moderate-exercise intensity. ^∗^*P* < .05.

Figure 2 depicts a representation of the $\dot{\text{V}}$O_2P_ kinetics obtained during the high-intensity bouts with and without BFR. The final $\dot{\text{V}}$O_2P_, HR, and Q_T_ kinetics analysis is shown in Table 2. The dynamics of $\dot{\text{V}}$O_2P_ and Q_T_ toward steady-state were slower during the moderate-intensity exercise with BFR ($\dot{\text{V}}$O_2P_ MRT: 41 ± 8 to 84 ± 17 seconds; and Q_T_MRT: 34 ± 10 to 80 ± 22 seconds, respectively) (Table 2) which did not change during the high-intensity with or without BFR, and including the HR kinetics.

**Table 2:** Kinetic parameters of $\dot{\text{V}}$O_2_, HR, and Q_T_ during moderate and high-intensity exercise tests with and without blood flow restriction (BFR).

	Trial 1		Trial 2	
Variables	MIE with BFR-	HIE	MIE with BFR+	HIE
$\dot{\text{V}}$O_2P_ (mL/min)				
Baseline	463 ± 126	535 ± 91	510 ± 72	541 ± 85
Amplitude	585 ± 165	1055 ± 204	535 ± 115^∗^	1021 ± 260
τ (s)	26 ± 16	27 ± 10	47 ± 20^∗^	25 ± 10
TD (s)	15 ± 5	14 ± 6	37 ± 13^∗^	13 ± 4
MRT (s)	41 ± 10	41 ± 8	84 ± 17	38 ± 8
HR (beats/min)				
Baseline	106 ± 11	108 ± 17	98 ± 13	95 ± 10^†^
Amplitude	84 ± 11	72 ± 23	51 ± 17^∗^	58 ± 21
τ (s)	60 ± 21	46 ± 25	57 ± 23	61 ± 25
Q_T_ (L/min)				
Baseline	5.1 ± 1.1	5.4 ± 1.1	3.9 ± 1.1^∗^	4.3 ± 1.1^†^
Amplitude	3.6 ± 1.8	3.9 ± 1.0	1.5 ± 0.7^∗^	2.4 ± 1.7
τ (s)	32.1 ± 8.5	34 ± 10	80 ± 22^∗^	40 ± 18
Time tolerance (s)	360	136 ± 30	360	220 ± 45^†^
Gross efficiency (%)		27.1 ± 5.4		32.8 ± 6.3^†^

### 3.3 Exercise tolerance time and efficiency

After the moderate-intensity exercise with BFR, exercise tolerance time increased in the subsequent bout of high-intensity exercise (136 ± 30 to 220 ± 45, *P* < .05) (Table 2). A significant increase in gross efficiency was also observed (27 ± 5.4 to 32.8 ± 6.3, *P* < .05) (Table 2 and Fig. 2).

## 4 Discussion

The primary purpose of the present study was to determine the effects of a priming moderate-intensity exercise associated with BFR on $\dot{\text{V}}$O_2_, HR and Q_T_ kinetics, time tolerance, and efficiency in a subsequent high-intensity exercise. To our knowledge, this is the first study to find that while a prior moderate-intensity exercise associated with BFR did not accelerate the $\dot{\text{V}}$O_2_, HR, and Q_T_ kinetics in a subsequent high-intensity exercise, it had significant improvements in both time tolerance and gross efficiency.

Several studies have used BFR training as a stand-alone method to improve subsequent exercise performance.^[10,30]^ The rationale is in accordance with previous study using a “PE”^[31]^ which demonstrated improvements in blood flow and oxygen delivery, which is essential for facilitating a speeding of the O_2_ kinetics. Studies on BFR^[32,33]^ have demonstrated that it produces an acute decline in oxygen delivery and metabolite clearance, reducing the availability of oxygen to the active musculature.

The occlusion phase may accelerate the pulmonary and cardiovascular (Q_T_) kinetics and enhance type II muscle fiber recruitment (lower blood flow/$\dot{\text{V}}$O_2_), which can be explained by the reduction in oxygen and subsequent metabolic accumulation of lactate during exercise,^[34]^ increasing muscle fiber recruitment via stimulation of group III and IV afferent pathways.^[35]^

In the current study, we observed that the use of BFR with a moderate-intensity exercise resulted in an acute and momentary slowness of $\dot{\text{V}}$O_2_ and Q_T_ kinetics, followed by unchanged $\dot{\text{V}}$O_2_, HR, and Q_T_ kinetics during the subsequent high-intensity exercise in comparison with the control study. Interestingly, we observed a substantial increase in time tolerance and gross efficiency during the high-intensity exercise. This result suggests that the addition of BFR to a priming moderate-intensity exercise may also stimulate other peripheral adaptations,^[9]^ including higher muscle recruitment and energy availability, improving efficiency. It is possible that higher muscle recruitment could be observed as the slow component of $\dot{\text{V}}$O_2_ increases. Unfortunately, we were not able to demonstrate this, possibly because study participants may have stopped the protocol before muscle fatigue happened, which takes time to develop and consequently demonstrate the slow component in the VO_2_ kinetics analysis.

As to the O_2_ kinetics and muscle deoxygenation, Kido et al^[36]^ found a significant change in muscle deoxygenation dynamics. However, the authors did not observe any difference in pulmonary $\dot{\text{V}}$O_2_ kinetics, with a similar protocol, using a 5-minute interval time between the vascular occlusion ischemic preconditioning and the actual exercise. Subsequently, Kilding et al^[37]^ also used a RIPC priming approach, analyzing its effects on high-intensity exercise. Whereas the authors did not demonstrate changes in phase II of the $\dot{\text{V}}$O_2_, there was a reduction in the $\dot{\text{V}}$O_2_ slow component and metabolic power expenditure, which is indicative of a beneficial priming effect.

Recently, Helal et al^[9]^ failed to demonstrate any influence of RIPC on $\dot{\text{V}}$O_2_, HR, and O_2_ pulse kinetics during a subsequent high-intensity exercise. The major difference between the studies mentioned above and the present study was that we used vascular occlusion associated with a priming moderate-intensity exercise, which has been recommended according to more recent reviews.^[38]^ The underpinning mechanism(s) explaining physiological changes following BFR in humans is, at present, poorly understood.^[37]^

Both cardiopulmonary and muscle responses have also been associated with increased vasodilatation response after reperfusion.^[39]^ Cruz et al^[12]^ suggested that hyperemia may improve the distribution of the regional skeletal muscle blood flow to the most active fibers, matching O_2_ demand with O_2_ delivery, or increasing mean capillary transit time, allowing for higher O_2_ extraction per unit of blood flowing through the fully activated muscle fibers. Thus, studies examining BFR training have shown an increase in the NO production capacity, accelerating glucose uptake by the exercising skeletal muscle,^[40]^ and promoting a more precise local matching of blood flow to metabolic rate. NO production seems to have a powerful effect on the reduction in $\dot{\text{V}}$O_2_ slow component amplitude changing of the recruitment type II muscle fibers, which are most active in high-intensity exercise.^[41]^ The loss of mechanic efficiency and fatigue of type II fibers are considered to be responsible for the higher occurrence of the $\dot{\text{V}}$O_2_ slow component,^[42]^ and, therefore, BFR may increase the metabolic efficiency of type II fibers, despite not influencing responses during moderate-intensity exercise since it mainly requires type I fibers,^[37]^ which can elucidate the potential muscle fiber type-specific effect of BFR on exercise tolerance.

We acknowledge some potential limitations of our study concerning the BFR administration and the inclusion of only female volunteers. Firstly, as in Cruz et al,^[12]^ we found difficulties in blinding the participants. Perhaps the main methodological constraint in BFR studies is the inability to blind subjects completely^[12,43]^ because the sensations induced by BFR vs the low-pressure protocol are different.^[44]^ Secondly, despite the care that crossover studies require and the care necessary to check the effectiveness of the results, it is not possible to rule out a potential placebo effect, although it is unlikely that individuals have managed their metabolism. Thirdly, we recognize that as the ideal time to reach maximum effects of BFR is not established, 15 minutes was an arbitrary value. Studies have reported different time intervals between priming and subsequent exercise bouts, which ranged from a few minutes^[45,46]^ to almost an hour.^[47,48]^ However, the application of short-time transitions might be able to potentiate the effects of reperfusion on the exercising skeletal muscle, increasing the circulation time and greater availability of O_2_. Additionally, although the inclusion of female subjects only may appear to be a limitation, we also believe that it might be considered a strength, considering the well-established sex discrepancies due to biological factors between men and women, regarding cardiopulmonary capacity and exercise tolerance. It is also essential to highlight the significant scarcity of scientific evidence in female populations. Menstrual cycle information of the study sample was not controlled since it seems not to influence cardiorespiratory capacity.^[49]^

## 5 Conclusions

This study found that a priming bout of moderate-intensity exercise associated with BFR did not accelerate the $\dot{\text{V}}$O2, HR, and Q_T_ kinetics assessed during a subsequent high-intensity exercise. However, significant improvements in both gross efficiency and exercise tolerance were observed when high-intensity exercise was performed following BFR training stimuli, without changes in cardiovascular performance.

## Acknowledgments

The authors would like to thank all colleagues from the Cardiorespiratory Rehabilitation Research Group (GPRC) for their collaboration and our patients for their effort and enthusiastic cooperation throughout the study. We also thank Prof Dr José Alberto Neder (Queens University, Canada) for encouraging this study rationale.

## Author contributions

**Conceptualization:** Robson F. Borges, Gaspar R. Chiappa, Paulo T. Muller, Lawrence Patrick Cahalin, Gerson Cipriano.

**Data curation:** Robson F. Borges, Gaspar R. Chiappa, Graziella França Bernardelli Cipriano, Gerson Cipriano.

**Formal analysis:** Robson F. Borges, Gaspar R. Chiappa, Paulo T. Muller, Lawrence Patrick Cahalin, Graziella França Bernardelli Cipriano, Gerson Cipriano.

**Funding acquisition:** Gaspar R. Chiappa, Gerson Cipriano.

**Investigation:** Robson F. Borges, Gaspar R. Chiappa, Paulo T. Muller, Alexandra Correa Gervazoni Balbuena de Lima, Graziella França Bernardelli Cipriano, Gerson Cipriano.

**Methodology:** Robson F. Borges, Gaspar R. Chiappa, Paulo T. Muller, Lawrence Patrick Cahalin, Graziella França Bernardelli Cipriano, Gerson Cipriano.

**Project administration:** Robson F. Borges, Gaspar R. Chiappa, Gerson Cipriano.

**Resources:** Graziella França Bernardelli Cipriano, Gerson Cipriano.

**Validation:** Alexandra Correa Gervazoni Balbuena de Lima, Gerson Cipriano.

**Visualization:** Alexandra Correa Gervazoni Balbuena de Lima, Gerson Cipriano.

**Writing – original draft:** Robson F. Borges, Gaspar R. Chiappa, Paulo T. Muller, Lawrence Patrick Cahalin, Graziella França Bernardelli Cipriano, Gerson Cipriano.

**Writing – review & editing:** Robson F. Borges, Gaspar R. Chiappa, Paulo T. Muller, Lawrence Patrick Cahalin, Graziella França Bernardelli Cipriano, Gerson Cipriano.
